# Examination of the Relationship between In-Store Environmental Factors and Fruit and Vegetable Purchasing among Hispanics

**DOI:** 10.3390/ijerph14111305

**Published:** 2017-10-27

**Authors:** Jennifer Sanchez-Flack, Julie L. Pickrel, George Belch, Shih-Fan Lin, Cheryl A. M. Anderson, Maria Elena Martinez, Elva M. Arredondo, Guadalupe X. Ayala

**Affiliations:** 1 Health Behavior and the Institute for Behavioral and Community Health, San Diego State University, University of California at San Diego Joint Doctoral Program in Public Health, 5500 Campanile Drive, San Diego, CA 92182, USA; 2 Institute for Behavioral and Community Health, San Diego State University Research Foundation, 9245 Sky Park Court, Suite 220, San Diego, CA 92123, USA; jpickrel@mail.sdsu.edu (J.L.P.); slin@mail.sdsu.edu (S.-F.L.); 3 Marketing Department, Fowler College of Business, San Diego State University, 5500 Campanile Drive, San Diego, CA 92182, USA; gbelch@mail.sdsu.edu; 4 Family Medicine and Public Health, University of California, San Diego, 9500 Gilman Drive, #0725, La Jolla, CA 92093, USA; c1anderson@ucsd.edu; 5 Family Medicine and Public Health, University of California, San Diego, 9500 Gilman Drive, #0901, La Jolla, CA 92093, USA; e8martinez@ucsd.edu; 6 Graduate School of Public Health, San Diego State University and the Institute for Behavioral and Community Health, 9245 Sky Park Court, Suite 220, San Diego, CA 92123, USA; earredon@mail.sdsu.edu (E.M.A.); ayala@mail.sdsu.edu (G.X.A.)

**Keywords:** consumer food environment, Latinos/Hispanics, store audits

## Abstract

Retail food environments have received attention for their influence on dietary behaviors and for their nutrition intervention potential. To improve diet-related behaviors, such as fruit and vegetable (FV) purchasing, it is important to examine its relationship with in-store environmental characteristics. This study used baseline data from the “*El Valor de Nuestra Salud*” study to examine how in-store environmental characteristics, such as product availability, placement and promotion, were associated with FV purchasing among Hispanic customers in San Diego County. Mixed linear regression models indicated that greater availability of fresh FVs was associated with a $0.36 increase in FV purchasing (*p* = 0.01). Placement variables, specifically each additional square foot of display space dedicated to FVs (*p* = 0.01) and each additional fresh FV display (*p* = 0.01), were associated with a $0.02 increase and $0.29 decrease, respectively, in FV purchasing. Introducing FV promotions in the final model was not related to FV purchasing. Exploratory analyses indicated that men reported spending $3.69 fewer dollars on FVs compared to women, controlling for covariates (*p* = 0.02). These results can help inform interventions targeting in-store environmental characteristics to encourage FV purchasing among Hispanics.

## 1. Introduction

Retail food environments, such as grocery and other food stores, have received increased attention for their influence on dietary behaviors and for being places to promote healthful eating [1]. These environments are situated between individuals and the foods and beverages they consume, making them an opportune setting to promote healthy dietary behaviors and prevent and control obesity [2].

Typically, the relationship between the retail food environment and dietary behaviors is studied in one of two ways: by examining the neighborhood environment (e.g., density of food stores in a census tract) or by examining the in-store environment (e.g., availability of items in a food store) [3,4].

Glanz et al.’s [3] model of community nutrition environments considers the nutrition environment from an ecological perspective, identifying four types of nutrition environments that need to be studied, including the in-store environment. Characteristics of the in-store environment important to the study of dietary behaviors include the availability and promotion of healthy and unhealthy foods and beverages; these can have an indirect or direct influence on what is purchased [3]. Likewise, Rose et al.’s multi-dimensional conceptual model proposes that in-store food availability, including shelf space, influences consumers’ purchasing behaviors [4]. Additionally, social cognitive theory proposes a reciprocal relationship between environmental factors and personal characteristics to explain behavior, with environmental factors representing situational influences (e.g., availability of healthy or unhealthy foods) on behaviors, such as purchasing [5]. These models, in conjunction with key elements of the marketing mix (i.e., product availability, placement and promotion) [6], were used as frameworks to examine the relationship between in-store environmental characteristics and fruit and vegetable (FV) purchasing.

### 1.1. In-Store Characteristics and Behaviors: Intake and Purchasing

Numerous studies in marketing research have shown that the in-store environment affects customers’ dietary behaviors. Historical research has demonstrated that the amount of shelf space [7], number of displays in a store and the number of locations an item was found in a store [8,9], as well as in-store advertising and promotions [10,11], influence customers’ purchasing of foods and beverages. For example, Curhan [7] found that doubling display space for fruit increased sales by 44%. Such findings have encouraged public health research on the relationship between in-store environmental characteristics and dietary behaviors [12,13].

With regard to the study of product availability, two studies found that the likelihood of purchasing FVs was higher among non-Hispanic blacks, non-Hispanic whites and Hispanics when there was a greater variety of FVs available in stores [14,15]. Similarly, a longitudinal study found that non-Hispanic white individuals who lived in communities with more varieties of FVs in stores had greater increases in weekly servings of FVs consumed over a one year period than individuals who lived in communities with fewer varieties of FVs [16]. In New York City, the association between the likelihood of purchasing a sugar-sweetened beverage decreased with greater availability of FVs located at the front of a store [15]. Other studies have not found a significant relationship between availability of FVs in stores and intake of FVs [17,18,19].

Research has also examined the placement of food in retail stores, specifically shelf or display space. One study found an association between the proportion of total shelf space in a store dedicated to red meat, reduced fat-milk and non-white bread, and intake of these foods among 12 communities in California and Hawaii [20]. Similarly, a strong positive relationship was observed between proportion of total shelf space in a store dedicated to low-fat milk and the prevalence of low-fat milk intake among a predominantly non-Hispanic white sample [21]. Recently, researchers observed that each extra meter of shelf space dedicated to vegetables was associated with an additional intake of 0.35 servings of vegetables per day among non-Hispanic white and non-Hispanic black residents living in New Orleans, LA, USA [22]. However, no significant relationship was found between shelf space dedicated to F and F intake [22].

Research on in-store displays found that each additional display location for alcoholic beverages, sugar-sweetened beverages and coffee in a store was associated with greater sales of these beverages [23]. Similarly, a longitudinal study found that individuals in communities with stores that have more FV displays showed greater intakes of these foods compared to those living in communities with stores having fewer FV displays [16]. However, a study conducted in Pittsburgh, PA, found no association between exposure to displays of sugar-sweetened beverages, snack foods and nutritious foods, and intake of sugar-sweetened beverages and FVs [24].

Regarding promotions, limited research has examined the influence of print promotions or signage (e.g., flyers, posters, banners, etc.) in stores; most previous research relates to television advertising [25,26]. Research conducted among adolescents found that frequent exposure to alcohol promotions in stores was associated with a 50% increase in the likelihood of ever drinking [27]. Additionally, a study conducted in New York City found that stores were more likely to display sugary drinks promotions in neighborhoods with higher intakes of sugar-sweetened beverages compared to stores in neighborhoods with lower intakes of sugar-sweetened beverages [28]. Lastly, among low-income public housing residents, higher counts of alcohol print promotions and lower counts of low-calorie food print promotions in stores and restaurants were associated with higher dietary fat intake [29].

### 1.2. Importance of Studying Racial/Ethnic Groups

Given the evidence supporting the relationship between in-store environmental characteristics and dietary behaviors, additional research is needed within specific understudied racial/ethnic groups, including Hispanics. Although Hispanics have been shown to purchase more FVs than non-Hispanic blacks [30], Hispanics are not meeting recommended dietary guidelines for FVs, consistent with other racial/ethnic populations [31]. The current USDA dietary guidelines recommend U.S. adults consume 1.5–2 cup equivalents of fruits (e.g., one cup equivalent = one small apple) and 2–3 cup equivalents of vegetables (e.g., one cup equivalent = 12 baby carrots) daily [32,33]. Recent estimates from the National Health and Nutrition Examination Survey (NHANES) indicate that the median cup equivalent intakes for Hispanics are 0.78 for fruits and 1.33 for vegetables [31]. Understanding how in-store environmental characteristics are associated with Hispanics’ FV purchasing is important for identifying ways to improve their dietary intake.

Hispanics in Southern California have been shown to shop in Hispanic-focused grocery stores, otherwise known as *tiendas* [34]. These *tiendas* offer a variety of high-quality and affordable FVs. In fact, one study demonstrated that *tiendas* offered FVs at a lower cost than supermarkets in the same region; this resulted in savings of over $U.S. 3/week for a diet of 2000 kcal/day [35]. The evidence that FVs in *tiendas* may be offered at lower prices than supermarkets suggests that price alone is not the driving factor in Hispanics’ low FV intake. Therefore, studying other in-store environmental characteristics in *tiendas*, such as availability of and display space dedicated to FVs, may provide valuable insight into the role that the in-store environment plays in Hispanics not meeting recommended dietary guidelines for FVs.

### 1.3. Present Study

Modifiable and strategic elements of the marketing mix, specifically product availability, placement and promotion, were used as the overarching conceptual framework. Price served as a covariate in the models given challenges with its modifiability [36]. Operationalizations of these marketing mix elements were as follows: (1) product availability: the availability of fresh, canned, and frozen FVs and the variety of fresh FVs; (2) placement: number of fresh FV displays and measured display space dedicated to fresh FVs; and, (3) promotion: number of FV promotions, including signage, and number of cross-product category promotions, which refers to promoting a product category in conjunction with a complementary product category (e.g., bananas promoted at cereal displays) [2]. Using baseline data from the “*El Valor de Nuestra Salud*” (The Value of Our Health) study, the present study examined the relationships between in-store environmental characteristics and dietary behaviors. It was hypothesized that each additional marketing mix element would enhance the explanatory value of the elements of the marketing mix on customers’ self-reported FV purchasing, adjusting for fresh FV prices. This study fills a gap in the literature by contributing unique information on the in-store environment of Hispanic-focused grocery stores and how Hispanic customers’ FV purchasing is influenced by these stores’ environmental characteristics.

## 2. Materials and Methods

### 2.1. Data Source

Baseline data were collected in the “*El Valor de Nuestra Salud*” study, a cluster randomized controlled trial (RCT) with 16 tiendas in San Diego County, California, USA. San Diego County is located on the U.S.-Mexico border where approximately 33% of the population is of Hispanic origin [37].

*Tiendas* were systematically sampled following an extensive enumeration process begun in June 2010. The systematic enumeration was conducted using five sources: (1) county food permits, (2) the county health department directory of food retailers, (3) the Special Supplemental Nutrition Program for Women, Infants, and Children (WIC) program active vendor list, (4) the Supplement Nutrition Assistance Program (SNAP) authorized retailer list, and (5) a previous observational study conducted in the target area [38]. Duplicates, non-food stores, and stores identifiable as not a *tienda* through internet and phone verification (e.g., super centers, liquor stores, etc.) were removed. Next, ZIP codes where the 2000 U.S. Census data indicated that the proportion of Hispanic residents was less than 20% were excluded in addition to San Diego’s South County because of competing intervention activities, leaving 566 entries in the enumeration list. Initial phone and internet verification activities reduced the list to 339.

Given time and resource constraints during recruitment, the study team identified four additional ZIP codes near the study offices that contained census tracts representing at least a 20% Hispanic population, based on updated 2010 Census data. From these areas, additional entries were added to the previously enumerated list of possible *tiendas*, which resulted in 382 entries available for verification. Store screening assessments were conducted to determine if stores met the operational definition of a *tienda* and other eligibility criteria: largely Hispanic customer-base; some or all employees were bilingual (English/Spanish language) or Spanish-speaking; some or all in-store product signage and promotions were bilingual and/or Spanish language; offered products/services from Mexico or other Latin American countries. In addition, *tiendas* were required to have a service butcher and fresh FV department; full service supermarkets were excluded. Of the 382 entries left on the enumeration list, plus an additional 26 stores identified during ground truthing, 273 were not eligible and six (1.5%) were duplicates, leaving 129 in the recruitment pool. From among the final list of *tiendas*, 84 were approached for participation, 14 were not approached for other exclusionary reasons (e.g., owned by participating owner; proximity to another participating store) and 31 were not approached given that we met our recruitment goal of 16 stores by September 2013. *Tiendas* were identified that were located at least one mile away from each other to minimize the potential for cross-contamination during the parent trial. Power calculations to determine the number of stores needed were based on parent study goals for the clustered randomized controlled trial (see Ayala et al. [39]). The target sample size was 16 stores and 23 customers per store. At baseline, the 16 *tiendas* were pair-matched on store size and the presence or absence of a prepared foods service department. Store owners/managers received monetary incentives for participating in interviews like those conducted with customers (see below), and stores that provided monthly sales data received $75 for each month the data were provided. Stores randomized to the intervention condition received intervention materials and employee trainings, and stores randomized to the wait-list control condition were offered materials and employee trainings after they completed all assessments. At baseline, stores knew that the intention of the parent study was to promote healthy foods, including FVs, but did not know that FVs were the primary target of the study.

Hispanic customers were recruited from these *tiendas* to participate on an evaluation cohort. Among the eligibility criteria for participation were: self-identified as Hispanic/Latino; 18 years of age or older; visited the *tienda* at least once a week to purchase food and beverages; purchased at least 50% of the groceries for his/her household at the recruited *tienda*; did not grocery shop at another study *tienda*; no dietary restrictions on consumption of FV; consumed four or fewer cups of FVs per day; able to read in Spanish; and planned to remain in the area for the one-year study duration. Only one participant per household could participate to minimize interdependent data. Following the eligibility screening and informed consent processes, participants took part in a 60-minute in-person interview and received $15. Interviews took place immediately after recruitment at the store or were scheduled for a later time at a location convenient for the customer (such as the customer’s home or a nearby community center or park). The interview consisted of the administration of dietary screeners, assessment of psychosocial, socio-cultural, demographic characteristics and measurement of weight. A total of 6488 customers were approached for participation; 4270 (66%) refused to be screened for eligibility upon approach and three (0.05%) requested to be screened by phone but attempts to reach these customers by phone were unsuccessful, leaving 2215 (34%) customers screened for eligibility. Of those who were screened, 1259 (57%) were deemed ineligible and eight (0.36%) had unknown eligibility (e.g., refused or incomplete screening forms). Of the 948 eligible customers, 24 (3%) were identified as ineligible before or during the interview (e.g., lived in the same household as another participant, may relocate out of area within study period), 239 (25%) customers declined to participate and nine (0.95%) were dropped due to incomplete baseline data or because the store was dropped from participation. The study completed recruitment and baseline data collection while 307 (32%) were still in the recruitment process, so they were no longer pursued. Our final sample size was 369 customers (*n* ≅ 23/store to ensure a balanced design for the RCT).

Audit data of the in-store FV environment were collected by five trained evaluators, including the project manager and evaluation coordinator, between November 2011 and October 2013. Audits occurred at varying times of day, Monday through Friday. To avoid potential social desirability bias, store owners/managers were unaware of what specific days or times evaluators would be conducting the audits. In addition, they were unaware that the primary focus was on the promotion and availability of FVs during these observations. Store audits collected data on availability of fresh, canned, frozen and prepared FVs, variety of fresh FVs, price of fresh FVs, display space dedicated to fresh FVs, number of fresh FV displays and FV promotions. To assess inter-rater reliability, 100% of baseline store audit data were collected by two blinded evaluators at the same time. Additional details on the “*El Valor de Nuestra Salud*” study procedures are described elsewhere [39]. This study was approved by the Institutional Review Board at San Diego State University (731084).

### 2.2. Outcome Measure: FV Purchasing

During interviews, participants were asked: “In a typical week, about how much do you spend on FVs?” and “You said that in a typical week you spend about $ (answer provided in previous question) on FVs. How much of this was spent here at THIS store?”. “THIS store” refers to the *tienda* from which the participant was recruited. For the purposes of this study, the outcome variable of interest is participants’ self-reported dollars spent on FVs at the “*El Valor de Nuestra Salud*” *tienda* (continuous). This methodology for collecting self-reported spending has been used in previous studies and in U.S. national surveys such as NHANES [40,41].

### 2.3. In-Store Environmental Characteristics: Product Availability

#### 2.3.1. Availability of Fresh, Canned and Frozen FVs

A store audit was conducted to assess the availability of fresh, canned and frozen FVs. Data on the availability (categorical: yes (coded as “1”); no (coded as “0”)) of fresh, canned, and frozen FV types were collected for a predetermined list, based on previous evidence, of 73 fresh FVs, 16 frozen FVs and 28 canned FVs, including mixed Fs and mixed Vs as a category for each [42,43,44], plus any other FVs present. In the current study, availability was defined as follows: (1) the total number of fresh FVs (e.g., apple, banana, avocado and carrots); (2) the total number of frozen FVs (e.g., strawberries and broccoli); and (3) the total number of canned FVs (e.g., applesauce and beets). Availability scores were computed by summing the available fresh FVs, frozen FVs, and canned FVs (continuous), respectively [45]. However, given the high correlation between canned FVs and frozen FVs (r = 0.803), these two variables were summed to create a single score of total number of canned and frozen FVs (continuous).

#### 2.3.2. Fresh FV Variety

Store audits also assessed the variety of fresh FVs stocked within a *tienda* for each type of fresh FV available. For example, if apples were stocked within the *tienda*, the number of unique varieties of apples were counted (e.g., gala, red delicious, granny smith and fuji apples). A total variety score was computed by summing the total number of FV varieties combined (all continuous) [22]. A strong correlation between availability of fresh FVs and varieties of fresh FVs (r = 0.974) was found, and therefore, varieties of fresh FVs were not included the model building process.

### 2.4. In-Store Environmental Characteristics: Placement

#### 2.4.1. Fresh FV Displays

Data were collected on the number (categorical: present (coded as “1”); not present (coded as ”0”)) and type (categorical: one-sided, pallet, island, promotion and other) of fresh FV displays using a “produce display tracking form” developed by the study team. Displays that only stocked prepared or cooked FVs were not counted (e.g., prepared salads). To capture the number of FV displays present, a variable was computed summing the total number of displays present at baseline for each *tienda* (continuous) [46].

#### 2.4.2. Display Space Dedicated to Fresh FVs

A “produce display tracking form”, developed by the study team, was used to assess the amount of display space dedicated to fresh FVs. Data on the measurements of shelves for each fresh FV display (continuous: length and width in feet) and level of stock (categorical: >0–1/3 full, >1/3–2/3 full, >2/3-completely full) within the display were collected. If the display contained items that were not fresh FVs, the length and width for these areas were also recorded and later subtracted to obtain an accurate measurement of display space solely dedicated to fresh FVs. All measurements were rounded to the nearest inch and then recorded in feet. Displays that only stocked prepared or cooked FVs were not measured (e.g., fruit salad with yogurt). To determine the total amount of display space dedicated to fresh FVs, a variable was computed summing display measurements for all FV displays in the *tienda* (continuous) [47].

### 2.5. In-Store Environmental Characteristics: Promotions

#### FV Promotions

Promotions of fresh, canned and frozen FVs were assessed using a “fruit and vegetable promotions form”, which captured detailed information on materials used to promote FVs within *tiendas* [48]. Data collected assessed the location of promotions (categorical: outside of store, aisles, checkout, endcaps, entrance, island, edge or other open space), product category of the item closest to the promotion (categorical: fresh FV, cereal and breakfast foods, snack foods, sugar-sweetened beverages, grains and dried beans, canned foods and soups (including canned FVs), dairy, butcher, frozen foods (including frozen FVs) alcoholic beverages, prepared foods, deli, bakery, tortillas, other grocery, non-food and other), and the type of promotion (categorical: price promotions, signage, handout, package add-on, theme and other) and number of promotions (continuous). Similar to previous research examining the influence of promotion exposure on dietary behaviors, the total number of FV promotions present was summed for each *tienda* (continuous) [29]. Given the influence of cross-product marketing on purchasing, a second variable was created to identify promotions found in cross-product category locations (dichotomous: FV promotion adjacent to fresh FVs versus anything else) [49]. A variable reporting the total number of “cross-product category” FV promotions within each *tienda* was computed by summing the number of FV promotions that were adjacent to a product category other than fresh FV (continuous). However, given the strong positive correlation between cross-product category promotions and number of FV promotions (r = 0.967), cross-product category promotion location was not included in the model building process.

### 2.6. In-Store and Customer Characteristics: Covariates

#### 2.6.1. Store Size and Price of Fresh FVs

Given the association between store size and in-store environmental characteristics such as the availability of foods [50,51,52,53], the current study initially considered sales floor square footage (continuous) as a covariate in the model building process. However, given the strong correlation between store size and display space dedicated to fresh FVs (r = 0.805), store size was not included in final models. Analyses were adjusted for the price of fresh FVs given the relationships observed between price of FVs, purchasing and intake [16,54,55]. During the store audits, evaluators collected data on the current price for a pre-determined list of preferred fresh FVs available in the *tienda*; if the preferred variety of a particular FV was not available or priced, an alternative was identified. Price data were collected as “price per pound(s) (lb)” or “price per unit(s)” depending on how the *tienda* priced the fresh FVs. When prices were not posted per lb, estimated weights were derived using standard food weights from the U.S. Department of Agriculture national nutrient database for standard reference [56]. Standard food weights are provided in grams but were converted to lbs for the current study. If the weight was not available through the database, three samples of the item were weighed within a store and the average was used. Price per unit was converted to price per lb by dividing an item’s unit price by its typical weight ((price per unit)/(lbs per unit)) [19]. A store-level mean price for all fresh FVs was computed for each *tienda* (continuous) [57].

#### 2.6.2. Customer Characteristics

The following customer characteristics were considered in the model building process given previous evidence supporting the association between individual characteristics, socioeconomic status, acculturation and dietary behavior, including food purchasing [58,59,60]: age (continuous) [61,62]; gender (categorical: female or male) [61,62]; education (categorical: high school graduate, 7th–11th grade, 6th grade or less) [61,62]; marital status (categorical: married or living together as married, or not married) [61,62]; poverty threshold according to the U.S. Census Bureau poverty threshold in 2013 using reported income and household size data (categorical: above poverty level or below poverty level) [61,62]; food assistance program participation (categorical: participating in WIC and/or SNAP, or does not participate in WIC or SNAP) [61,62]; household size (continuous) [61,62], and generation status (categorical: born in U.S., born outside of U.S.) [63]. Length of time in the U.S. was considered but due to missing data among the U.S. born, it was not included in the final models.

### 2.7. Statistical Analyses

Analyses were performed using SAS software, Version 9.4 of the SAS System for Windows (SAS Institute, Cary, NC, USA). Descriptive statistics on FV purchasing, all in-store environmental characteristics and covariates were obtained. To assess inter-rater reliability of store audit data, Cohen’s kappa statistics were computed for binary variables [64] and intraclass correlations (ICCs) were computed for continuous variables [65] for a random sample of over a third of baseline store audits with reliability data. The inter-rater reliability analyses presented are for baseline data only.

A series of bivariate analyses were conducted to assess the unadjusted relationship between customer characteristics and FV purchasing. Customer characteristics with a *p* < 0.20 were included in the final model to control for sources of variance. These variables included: gender, marital status, household size, poverty status, and generation status based on place of birth. Prior to estimating the final mixed models, tests for multicollinearity among all in-store characteristics and identified customer characteristics from bivariate analyses were examined to assess for linear relationships among independent variables. Variables with variance inflation factors greater than 10 were examined further using Pearson’s correlation coefficients. Highly correlated variables were excluded in the final mixed models to avoid the problem of multicollinearity.

Given the normal distribution of customers’ self-reported FV purchasing and the data structure of customers nested in 16 *tiendas*, a linear regression model was estimated using PROC MIXED with a random statement to account for the nested structure within each *tienda*. The mixed models were estimated under the conceptual framework of the marketing mix elements with product availability variables entered first, placement variables second and promotion third. The order in which variables were entered into the models reflects the stores’ experience in acquiring FVs, merchandising FVs in the store and finally, promoting them to customers [44]. Therefore, model 1 estimated the association between product availability and FV purchasing, adjusting for FV price and customer characteristics. Model 2 introduced placement variables and estimated the association between product availability, placement, and FV purchasing, adjusting for covariates. Model 3 introduced promotion variables and estimated the association between product availability, placement, promotion and FV purchasing, adjusting for covariates.

## 3. Results

### 3.1. Inter-Rater Reliability Analyses

Across all in-store environmental characteristics, kappa coefficients and ICCs demonstrated moderate to perfect agreement for the audit data analyzed at baseline. At baseline, kappa coefficients were above 0.80–1.00 for product availability (availability of canned and frozen FVs) variables indicating moderate to perfect agreement [66]. ICCs ranged from 0.97 to 1.00 for product availability and variety of fresh FVs, placement (number of fresh FV displays and display space dedicated to fresh FVs) and promotion (number of FV promotions) variables indicating excellent agreement between evaluators [65].

### 3.2. Customer, Tienda and In-Store Environmental Characteristics

Descriptive characteristics of customers are presented in Table 1. More than two thirds (70%) of the sample was female with a mean age of 42 years. Most customers (88%) were born outside of the U.S. and 28% lived above the poverty threshold with a mean household size of about five. About 36% of customers were high school graduates and 60% reported being employed full-time, part-time or seasonally. Approximately half reported participating in a food assistance program(s). The mean reported weekly dollars spent on FVs from all sources was $36.13 (SD = $20.43) and the mean reported weekly dollars spent on FVs at the *tienda* was $16.41 (SD = $13.77).

Descriptive characteristics of *tiendas* and of the in-store environmental characteristics are presented in Table 2; means and medians are presented given the wide ranges observed. In terms of store size, the median square footage of sales floor was 2508.08 (range = 648.38–12,639.43) and the median number of cash registers was three (range: 1–5). For product availability variables, the median number of fresh FVs available was 48 and the median number of canned and frozen FVs available was 28. In terms of placement, the median number of fresh FV displays available was nine (range = 2–36) and the median display space dedicated to FVs was 289 square feet (range = 125.28–860.44). Lastly, for promotions, the median number of FV promotions in *tiendas* was about four (range = 0–103). The large range in the number of FV promotions is because a couple of *tiendas* were larger and had experience using their own FV-related signage.

### 3.3. In-Store Characteristics and FV Purchasing

Results of the linear regression mixed models, estimating the adjusted relationship between in-store environmental characteristics and FV purchasing, are presented in Table 3. Results from model 1, which estimated the association between product availability variables and FV purchasing, indicated a significant positive relationship between the availability of fresh FVs and FV purchasing. Each additional fresh FV available was associated with an additional $0.36 spent on FVs, adjusting for FV price and customer characteristics. In model 2, each additional fresh FV display was associated with a $0.29 decrease in spending on FVs; however, each additional square foot of display space dedicated to FVs was associated with an additional $0.02 spent on FVs. In this same model, availability of fresh FVs became non-significant. Model 3 introduced the FV promotions variable; it was not significantly associated with purchasing. However, the two placement variables remained significant, demonstrating that greater numbers of fresh FV displays were associated with less FV purchasing, whereas increased display space dedicated to fresh FVs was associated with greater FV purchasing. In all three models, there was a significant association between gender and FV purchasing. Compared to women, men reported fewer dollars spent on FVs, even after adjusting for all in-store environmental characteristics and other customer characteristics.

## 4. Discussion

This study examined the relationship between in-store environmental characteristics and FV purchasing among Hispanics who are customers of *tiendas* in San Diego County, CA, USA. We found that availability of fresh FVs was significantly associated with FV purchasing; however, after controlling for the influence of the number of fresh FVs displays and display space dedicated to FVs, this relationship was no longer significant. The number of FV promotions was not significantly associated with FV purchasing. Additionally, it was found that men reported fewer dollars spent on FVs compared to women, irrespective of in-store environmental characteristics.

These findings support previous research which found a positive relationship between display space dedicated to specific foods and purchase behavior for healthy foods [21,22,67]. Similar to the present study, Bodor et al. [22] found that the amount of display space dedicated to fresh vegetables was a significant and positive predictor of vegetable intake. However, the negative association between number of fresh FV displays and FV purchasing in our study is surprising. A possible explanation for this finding may be due to the vast number of foods available within a store. The number of fresh FVs displays within a *tienda* may reflect an overabundance of displays for all foods, which may be creating an over-stimulating environment for customers that hinders their purchasing decisions [68]. Previous research suggests that having too many choices within one product category may result in customers not choosing any item within that product category or being less satisfied with what they choose [69].

Another finding consistent with previous research is the relationship between fresh FV availability and purchasing [14,15]; however, this association no longer persisted after the introduction of placement variables. Additionally, promotion was not related to FV purchasing in the last model, which is consistent with previous intervention research conducted among Hispanics that found no improvement in FV purchasing after intervention efforts targeting the promotion of FVs through print materials alone [70]. Although the availability and promotion of foods is important in determining purchasing behavior, the accessibility and prominence of these foods, as measured by display space, appears to have an important impact on purchasing behavior [45].

Significant associations were also found for gender and FV purchasing, with men reporting fewer dollars spent on FVs than women. This finding aligns with FV dietary intake findings indicating that men are less likely to meet dietary guidelines for FVs than women [71]. Previous research has demonstrated that men are less likely to shop for food with a grocery list, which is important to note given that shopping without a grocery list is associated with impulse purchases [72], which are often for unhealthy foods such as some sugary or salty snacks [73]. In fact, manufacturers of such foods pay retailers “slotting allowances” to obtain specific retail display and shelf spaces in stores that are known to increase sales [74,75]. Additionally, the promotion of unhealthy food may have overshadowed the promotion of fresh FVs in the stores. This may mean that men in the present study were susceptible to impulse purchasing of unhealthy foods versus FVs, despite the availability, placement and promotion of FVs in a *tienda*. A recent report from the Food Marketing Institute revealed that men conduct at least 50% of the grocery shopping for their households [76]. Given these findings, it is important to further understand their purchasing behaviors and ways to influence them to make healthier purchases.

### 4.1. Limitations and Strengths

There are several limitations and strengths worth noting. First, we only examined FV purchasing, and as such, inferences about actual consumption are not possible. Second, we are unable to generalize findings to the purchases of other foods such as sweet and savory snacks. Third, due to difficulties in obtaining codable receipt data, data on purchases of FVs were self-reported, which are subject to measurement and recall error. Also, FV purchasing was self-reported for a typical week so it may not represent customers’ actual FV purchasing. Furthermore, given that FV purchasing was self-reported by an individual shopper in the household, it may not account for the FV purchasing of other household members. Fourth, this study did not account for the number of children in the household and how this may influence purchasing behavior in regards to the in-store environment [77]. Fifth, although the analyses controlled for individual characteristics, the study did not consider factors such as product knowledge and attitudes towards purchasing and consuming FVs [78]. Sixth, the type of fresh FV display was not considered, therefore the analyses did not account for differences in types of displays, such as end-caps versus islands, and their influence on FV purchasing [68]. Seventh, data collected on the number of fresh FV displays does not reflect number of exposures throughout the store to FVs; some displays counted could have been clustered together versus spread throughout the store. Eighth, although stores were matched in size for randomization purposes for the parent study, they varied in size and the store sample size is too small to test for differences by size. Lastly, analyses were based on cross-sectional data, therefore causality cannot be determined.

Strengths of the study include the use of objective measurements of the in-store environment. Additionally, this study fills a research gap as it focused on Hispanic shoppers of *tiendas*, a racial/ethnic group and retail food environment that have not been well represented in the literature in terms of FV purchasing.

### 4.2. Implications

Findings from this study have implications for practice and future research. As indicated, FV display space was significantly associated with FV purchasing, even after adjusting for product availability, promotion, price and customer characteristics. Although the parameter estimates for display space were small, more display space dedicated to FVs was associated with more dollars spent on FVs, whereas more fresh FV displays in a *tienda* were associated with fewer dollars spent on FVs. Therefore, from a merchandising perspective, expanding the amount of display space dedicated to FVs within existing displays may be more effective than increasing the number of FV displays as a strategy for increasing the purchasing of these foods.

In intervention research, this may mean utilizing choice architecture nutrition interventions. This type of nutrition intervention includes moving displays so they are immediately visible to customers and arranging shelves so that promoted products are located at eye level [79]. Such strategies have been shown to be successful for healthy food purchases, even among low-income Hispanic families [79,80]. In addition to increasing the visibility of FVs, it may be effective to decrease the visibility of unhealthy foods to minimize temptation. Developing intervention strategies that oversee the nutrient profile of foods placed in prominent locations in stores may mean increased purchases for healthy foods such as FVs [68]. Such interventions will need to address barriers to such changes resulting from slotting allowances paid by distributors of unhealthy foods and beverages [75].

This study also found that men reported significantly less money spent on FVs than women, even after accounting for all in-store environmental characteristics and customer characteristics. More research is needed on the food purchasing behaviors of men given the limited research available. Nutrition interventions targeting the purchasing behaviors of men are needed given that some are less likely to meet the dietary guidelines of FVs as compared to women and given that men have become more involved with household grocery shopping [76]. An in-store nutrition intervention may be effective for men given previous evidence suggesting their apprehensiveness of interpersonal interventions and receptiveness to worksite and community-based interventions [81].

## 5. Conclusions

Results suggest that amount of display space dedicated to FVs, even after accounting for product availability, promotion, price and customer characteristics, was associated with more dollars being spent on FVs. Longitudinal studies should examine the influence of product availability, placement and promotion on the purchasing of healthy and unhealthy foods and beverages. Future studies should continue to examine in-store environmental characteristics in unique food store environments, among other racial/ethnic populations and how exposure to different or multiple in-store environmental characteristics influence purchasing behavior.

## Figures and Tables

**Table 1:** “*El Valor de Nuestra Salud*” (The value of our health) customer characteristics (*n* = 369).

Customer Characteristics	Baseline *n* (%) or Mean (SD)	Missing *n* (%)
Age	42.18 (12.00)	
Female	259 (70.19%)	
Married or living as married	262 (71.00%)	
Above poverty threshold	102 (28.49%)	11 (3.00%)
Employed full-time, part-time or seasonal	223 (60.43%)	
Education		
6th grade or less	114 (30.89%)	
7th–11th grade	124 (33.60%)	
High school graduate or more education	131 (35.50%)	
Household size	4.70 (1.88)	
Participating in either the Supplemental Nutrition and Assistance Program (SNAP) or the Special Supplemental Nutrition Program for Women, Infants, and Children (WIC)	175 (47.55%)	1 (0.30%)
Foreign born	325 (88.08%)	
Years in the U.S. (among foreign born)	19.26 (9.97)	
Self-reported dollars spent on fruits and vegetables (FVs) in a typical week (all sources)	36.13 (20.43)	2 (0.54%)
Dependent variable		
Self-reported dollars spent on FVs at *tienda* in a typical week	16.41 (13.77)	3 (0.81%)

**Table 2:** “*El Valor de Nuestra Salud*” (The value of our health) baseline *tienda* and marketing mix characteristics (*n* = 16).

*Tienda* and Marketing Mix Characteristics	Baseline Mean (SD)	Baseline Median (Range)
Store size		
Number of cash registers	3.00 (1.41)	3.00 (1.00–5.00)
Number of aisles	4.56 (1.93)	4.00 (2.00–9.00)
Sales floor square footage	4083.35 (3694.33)	2508.08 (648.38–12,639.43)
Product		
Number of fresh FVs available	48.75 (9.33)	48.00 (32.00–63.00)
Variety of fresh FVs available	73.69 (20.83)	70.50 (42.00–115.00)
Number of canned and frozen FVs available	26.94 (12.07)	27.50 (7.00–46.00)
Placement		
Number of fresh FV displays	380.92 (229.17)	289.31 (1331.40–860.44)
Display space dedicated to fresh FVs (square feet)	11.69 (8.83)	9.00 (2.00–36.00)
Promotion		
Number of FV promotions (all types)	14.44 (25.95)	3.50 (0–103.00)
Number of cross-product category promotions	8.81 (18.68)	2.00 (0–76.00)

**Table 3:** Linear regression mixed models examining differences in the adjusted relationship between customer-reported FV purchasing and the introduction of each in-store environmental characteristics—product, placement, and promotion, *n* = 356 *.

Independent Variables and Covariates	Model 1			Model 2			Model 3		
	Beta (SE)	95% CI	*p*	Beta (SE)	95% CI	*p*	Beta (SE)	95% CI	*p*
Marketing mix									
*Product*									
Availability of fresh FVs	0.36 (0.13)	(0.09, 0.63)	0.01	0.11 (0.17)	(−0.25, 0.46)	0.52	0.12 (0.17)	(−0.25, 0.49)	0.50
Availability of canned and frozen FVs	0.07 (0.10)	(−0.14, 0.28)	0.48	−0.09 (0.09)	(−0.28, 0.10)	0.31	−0.09 (0.09)	(−0.28, 0.10)	0.33
*Placement*									
FV displays				0.02 (0.01)	(0.01, 0.04)	0.01	0.03 (0.01)	(0.01, 0.04)	0.01
Display space dedicated to FVs				−0.29 (0.11)	(−0.52, −0.06)	0.02	−0.30 (0.13)	(−0.57, −0.04)	0.03
*Promotion*									
FV promotions							−0.01 (0.05)	(−0.11, 0.09)	0.80
Adjustment variables									
*Customer Characteristics*									
Gender									
Female		Ref			Ref			Ref	
Male	−3.74 (1.54)	(−6.76, −0.72)	0.02	−3.60 (1.52)	(−6.59, −0.61)	0.02	−3.64 (1.53)	(−6.64, −0.63)	0.02
Poverty status									
Above poverty threshold		Ref			Ref			Ref	
Below poverty threshold	1.85 (1.56)	(−1.22, 4.93)	0.24	1.67 (1.55)	(−1.38, 4.71)	0.28	1.66 (1.55)	(−1.38, 4.71)	0.28
Household size	0.21 (0.38)	(−0.53, 0.96)	0.58	0.22 (0.38)	(−0.53, 0.96)	0.57	0.22 (0.38)	(−0.53, 0.97)	0.56
Marital status									
Not married		Ref			Ref			Ref	
Married/living as married	2.67 (1.60)	(−0.48, 5.82)	0.10	2.14 (1.61)	(−1.02, 5.29)	0.18	2.11 (1.61)	(−1.05, 5.27)	0.19
Foreign born									
No		Ref			Ref			Ref	
Yes	2.40 (2.16)	(−1.85, 6.65)	0.27	2.88 (2.16)	(−1.36, 7.13)	0.18	2.87 (2.16)	(−1.38, 7.12)	0.18
*Store characteristics*									
Price of fresh FVs (mean $s)	0.67 (4.55)	(−8.97, 10.30)	0.89	3.48 (3.86)	(−4.72, 11.67)	0.38	3.80 (4.05)	(−4.79, 12.40)	0.36

* 13 customers missing because of poverty and FV purchasing variables.
